# Expression of THSD7A in neoplasm tissues and its relationship with proteinuria

**DOI:** 10.1186/s12882-019-1489-5

**Published:** 2019-08-23

**Authors:** Li Xian, Dandan Dong, Jiamei Luo, Ling Zhuo, Ke Li, Ping Zhang, Wei Wang, Ying Xu, Gang Xu, Li Wang, Guisen Li

**Affiliations:** 1 Renal Division and Institute of Nephrology, Sichuan Academy of Medical Sciences and Sichuan Provincial People’s Hospital, School of Medicine, University of Electronic Science and Technology of China, No. 32, West 2nd Duan, 1st Circle Road, Qingyang District, Chengdu, Sichuan People’s Republic of China 610072; 2 Department of Pathology, Sichuan Academy of Medical Sciences and Sichuan Provincial People’s Hospital, No. 32, West 2nd Duan, 1st Circle Road, Qingyang District, Chengdu, Sichuan People’s Republic of China 610072

**Keywords:** Thrombospondin type 1 domain containing 7A, Membranous nephropathy, Proteinuria, Colorectal cancer, Breast cancer

## Abstract

**Background:**

Thrombospondin type 1 domain containing 7A (THSD7A) was recently identified target autoantigen in membranous nephropathy (MN). However, patients with positive THSD7A expression were prone to have malignancies. THSD7A was found to be expressed in a variety of malignant tumors. In this study, we investigated the histologic expression of THSD7A in colorectal or breast cancers, as well as the relationship between THSD7A expression and proteinuria in the patients with cancers.

**Method:**

A total of 101 patients were enrolled in the study, 81 of them had colorectal cancer and 20 had breast cancer. THSD7A expression was detected by immunohistochemical staining in tumor tissues. The clinical and laboratory parameters of these patients before their tumor resection were collected.

**Results:**

Positive expression rates of THSD7A in the two types of tumor tissues were very high, 97.5% in colorectal cancer, and 100% in breast cancer. THSD7A expression was also detected in lymph nodes of two patients with lymph node metastasis. Total 11 patients (10.9%) had proteinuria before surgery. Among the 4 patients who had proteinuria and were followed up, the proteinuria of 3 patients disappeared after surgery.

**Conclusions:**

The positive rate of THSD7A expression was very high in human colorectal cancer or breast cancer. It might be an important link between malignant tumors and kidney diseases.

**Electronic supplementary material:**

The online version of this article (10.1186/s12882-019-1489-5) contains supplementary material, which is available to authorized users.

## Background

Thrombospondin type 1 domain containing 7A (THSD7A) was recently identified as a new autoantigen in membranous nephropathy (MN) [1]. Similar to phospholipase A2 receptor (PLAR2), THSD7A is also a transmembrane glycoprotein with a molecular weight of 250 kDa [1–3]. It is expressed on the surface of the glomerular podocyte, and the predominantly corresponding antibody is IgG4 in patients with primary MN. While the antibody against THSD7A was isolated from serum of MN patients and injected into mice, proteinuria began to appear on the third day [4]. And renal biopsy showed that human anti-THSD7A antibodies specifically bounded to THSD7A on podocyte foot forming a typical histopathological pattern of MN [4]. The results demonstrated that THSD7A could induce the typical MN. Therefore, THSD7A might be a pathogenic autoantigen of MN [1, 4].

But the prevalence of THSD7A was relatively low, approximately 3–5% in idiopathic MN patients, and was predominantly expressed in PLA2R negative case [1, 5–9]. We have systemically reviewed the positive rate of THSD7A in patients with MN. A total of 4121 patients with MN from 10 studies were included in the analysis. The prevalence of THSD7A was 3% (95% CI, 3–4%) for all patients and 10% (95% CI, 6–15%) for PLA2R-negative patients [8].

Recently, a multicenter study demonstrated that 20% of MN patients with THSD7A positive had malignancies after diagnosis of MN [7]. Our analysis also showed that the prevalence of malignancy varied from 6 to 25% in THSD7A positive patients with MN [8]. It implied that patients with THSD7A-positive MN might be prone to have malignant tumors. Moreover, a patient with THSD7A positive MN was diagnosed with mixed adenoneuroendocrine carcinoma of the gallbladder, the positive expression of THSD7A was detected in the tumor tissues as well as the lymph nodes with the metastatic carcinoma [10]. Their further analyzed the relationship between malignancies and MN, the results showed that 7(28%) of all 25 THSD7A positive MN patients had a malignant tumor [10].

However, whether the patients with THSD7A-positive malignant tumors were more prone to kidney disease, and THSD7A is a bridge between malignant tumors and MN was still unclear. In this study, we investigated the histologic expression of THSD7A in colorectal or breast cancers, as well as the relationship between THSD7A expression and proteinuria in these patients.

## Methods

### Subjects

The adult patients (≥18 years old) with colorectal or breast cancer confirmed by pathological examination after surgery from November 2014 to July 2016 were included in the study. Inclusion criteria were as follows: newly pathological diagnosis of colorectal cancer or breast cancer; urinalysis performed before tumor resection and chemotherapy. Exclusion criteria were as follows: patients with previous diagnosis of chronic kidney diseases, diabetes mellitus, hypertension, or autoimmune diseases; patients with infection of hepatitis B virus, hepatitis C virus, or human immunodeficiency virus; patients with other infectious diseases, especially with urinary tract infection; patients with urinary calculi and urinary tract neoplasms; patients with acute kidney injury before surgery.

Finally, total 101 patients were enrolled in this study, 81 of them had colorectal cancer, and 20 had breast cancer. And 20 healthy individuals were enrolled as controls. This study was approved by Institutional Review Boards of the Sichuan Academy of Medical Sciences and Sichuan Provincial People’s Hospital. Written informed consents were obtained from all subjects.

### Immunohistochemistry (IHC)

Tissue samples (*n* = 101) from the Pathology Department of our hospital were fixed with formalin and embedded in paraffin. Tissue blocks were sliced into sections with the thickness of 3-μm. Subsequently the sections were dewaxed and rehydrated with xylene and alcohol. Then sections were exposed to heat-induced antigen retrieval for 3 min in an autoclave at 120 °C in citrate buffer (pH 6.0). Next the sections were incubated at the temperature of 4 °C overnight with rabbit anti-THSD7A (Sigma Aldrich, # HPA000923, dilution 1:150) and biotin-labeled EnVision Kit (Dako, #K5007, dilution 1:50) was added for 40 min. After being washed with PBS, samples were counter-stained with haematoxylin.

Two experienced pathologists blindly reviewed all tumor sections independently. The expression of THSD7A was determined by the staining intensity and the staining area. The score of staining intensity was categorized as follows: 0 was negative, one plus (1+) was weak positive, two plus (2+) was moderate, and three plus (3+) was strongly positive  (Additional file 2: Figure S1). The classification criteria of the positive staining area were defined as follows:0 indicated that the positive area was less than 5%, one plus (1+) indicated a positive area from 5 to 25%, and two plus (2+) was from 25 to 50%, three plus (3+) was more than 50%.

### Statistical analysis

THSD7A staining intensity and proteinuria status was measured by Semi-quantitative. Statistical analyses were performed by using SPSS 17.0 (SPCC Inc., Chicago, IL). All quantitative data conforming to normal distribution, were analyzed by the T test and ANOVA. Chi square test (Fisher’s exact test) and rank-sum test were used to qualitative data. Spearman’s rank-sum coefficient of correlation was used to analyze the correlation between proteinuria and THSD7A staining, and *P* value less than 0.05 was considered as being significant.

## Results

### Baseline characteristics of all patients

The characteristics of clinical and laboratory parameters of all the patients before surgery were listed in Table 1. The estimated glomerular filtration rate (eGFR) (96.5 ± 20.0 vs. 103.6 ± 17.5 ml/min/1.73m^2^, *P* = 0.006) and hemoglobin (111.9 ± 28.5 vs. 134.6 ± 15.3 g/L, *P* = 0.002) in colorectal cancer patients were significantly lower than those of control people. The difference in serum creatinine between the breast cancer group and the control group was significant (54.4 ± 9.9 vs. 62.5 ± 10.4 μmol/L, *P* = 0.021).

**Table 1:** Baseline characteristics of all subjects

	Colorectal cancer (*n* = 81)	Breast cancer (*n* = 20)	Control group (*n* = 20)	P1	P2
Gender (male/female)	54/27	0/20	7/13	0.01	0.008
Age (years)	59.0 ± 11.4	50.7 ± 11.3	52.7 ± 16.0	0.045	0.659
Scr (μmol/l)	66.4 ± 18.4	54.4 ± 9.9	62.5 ± 10.4	0.639	0.021
Bun (mmol/l)	5.6 ± 2.3	5.1 ± 1.3	4.7 ± 1.06	0.056	0.269
eGFR (ml/min/1.73m^2)^	96.5 ± 20.0	104.7 ± 15.1	103.6 ± 17.5	0.006	0.754
Alb (g/l)	38.7 ± 4.4	41.9 ± 6.7	41.7 ± 3.4	0.325	0.323
Hb (g/l)	111.9 ± 28.5	130.6 ± 13.0	134.6 ± 15.3	0.002	0.665

P1 is the *P* value of the comparison between colorectal cancer and control group; P2 is the *P* value of the comparison between cancer and control group; Scr, serum creatinine; BUN, blood urea nitrogen; Alb, serum albumin, eGFR, estimating glomerular filtration rate; Hb hemoglobin

### Expression of THSD7A in two types of malignant tumors

**Fig. 1:**
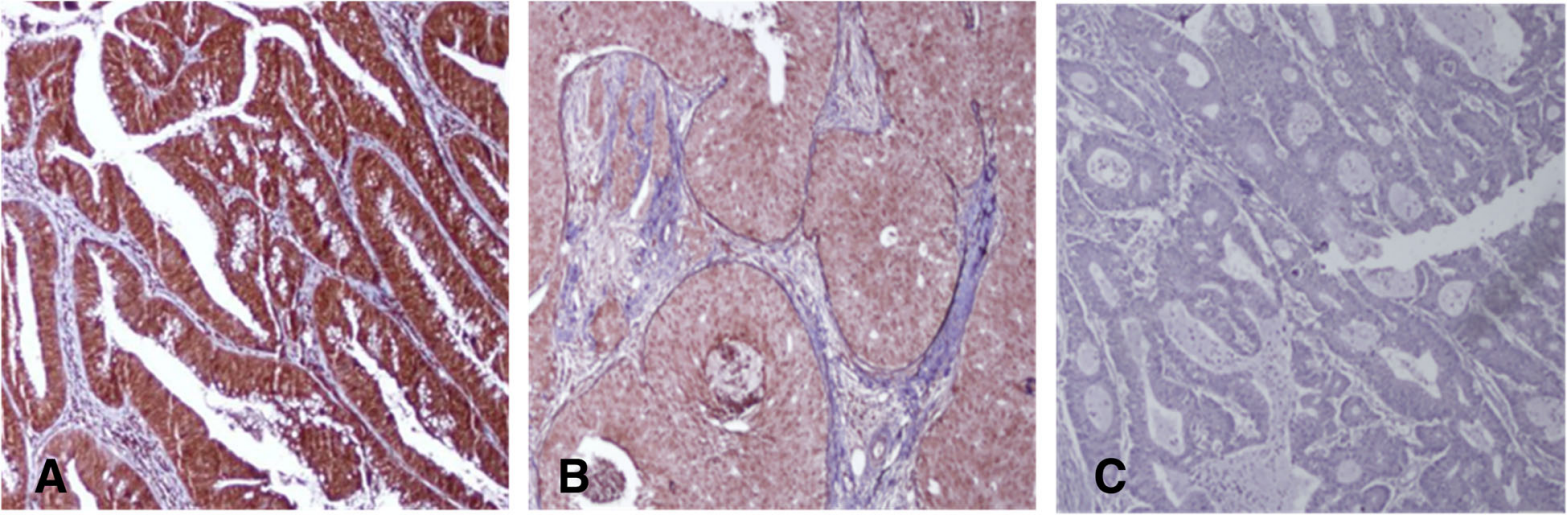
Expression of THSD7A in two cancer. **a**, colorectal cancer tissue with THSD7A staining positive; **b**, THSD7A positive in breast cancer tissue; **c**, THSD7A staining negative in colorectal cancer; THSD7A was expressed in the cell membrane and cytoplasm (**a** and **b**)

The results showed that THSD7A was positively expressed in colorectal cancer and breast cancer tissues by IHC staining. The positive expression of THSD7A was detected in colorectal and breast cancer sections, and THSD7A was expressed in the membrane and cytoplasm of tumor cells (Fig. 1). We analyzed both staining intensity and area of THSD7A. The results of staining intensity were shown in Table 2. Total 79 cases (97.5%) with colorectal cancer were THSD7A positive and most of them had strong positive expression of THSD7A. All 20 patients (100%) with breast cancer were THSD7A positive and most of them had moderate positive expression of THSD7A. The results of staining area were shown in Table 3, the area of positive staining was more than 50% in most patients with colorectal or breast cancer.

**Table 2:** Intensity of THSD7A staining in two cancers

THSD7A intensity	All (*n* = 101)	Colorectal cancer (*n* = 81)	Breast cancer (*n* = 20)
3+	69	62	7
2+	27	15	12
1+	3	2	1
Total /Per	99/(98%)	79/(97.5%)	20/(100%)

One plus (1+), weak positive; two plus (2+), moderate; three plus (3+), strong positive

**Table 3:** Area of THSD7A staining in two cancers

Area of THSD7A staining	All (*n* = 101)	Colorectal cancer (*n* = 81)	Breast cancer (*n* = 20)
3+	91	74	17
2+	8	5	3
1+	0	0	0
Total /Per	99/(98%)	79/(97.5%)	20/(100%)

One plus (1+), 5 to 25%; two plus (2+), 25 to 50%; three plus (3+), more than 50%

### Analysis of proteinuria in patients and its relationship with THSD7A

To decrease false positive rates, we excluded the proteinuria of 0.5+. Total 11 cases (10.9%) had positive proteinuria, 9 cases of them in colorectal cancer group (11%), and 2 cases in the breast cancer group (10%). And all with proteinuria were patients with tumor tissue THSD7A positive (Table 4). The detail information of 11 patients with positive proteinuria were shown in Additional file 1: Table S1. In addition, we analyzed the relationship between the proteinuria and the intensity as well as the area of THSD7A expression, the results showed there was no statistical correlation between proteinuria and expression patterns of THSD7A (*P* > 0.05) (Additional file 1: Tables S2–S5).

**Table 4:** Urinary protein in patients with cancer

Urinary protein	All (*n* = 101)	Colorectal cancer (*n* = 81)	Breast cancer (*n* = 20)	Control group (*n* = 20)
3+	1	1	0	0
2+	1	0	1	0
1+	9	8	1	0
Total/Per	11/(10.9%)	9/(11%)	2/(10%)	0/0

### Expression of THSD7A in lymph nodes

**Fig. 2:**
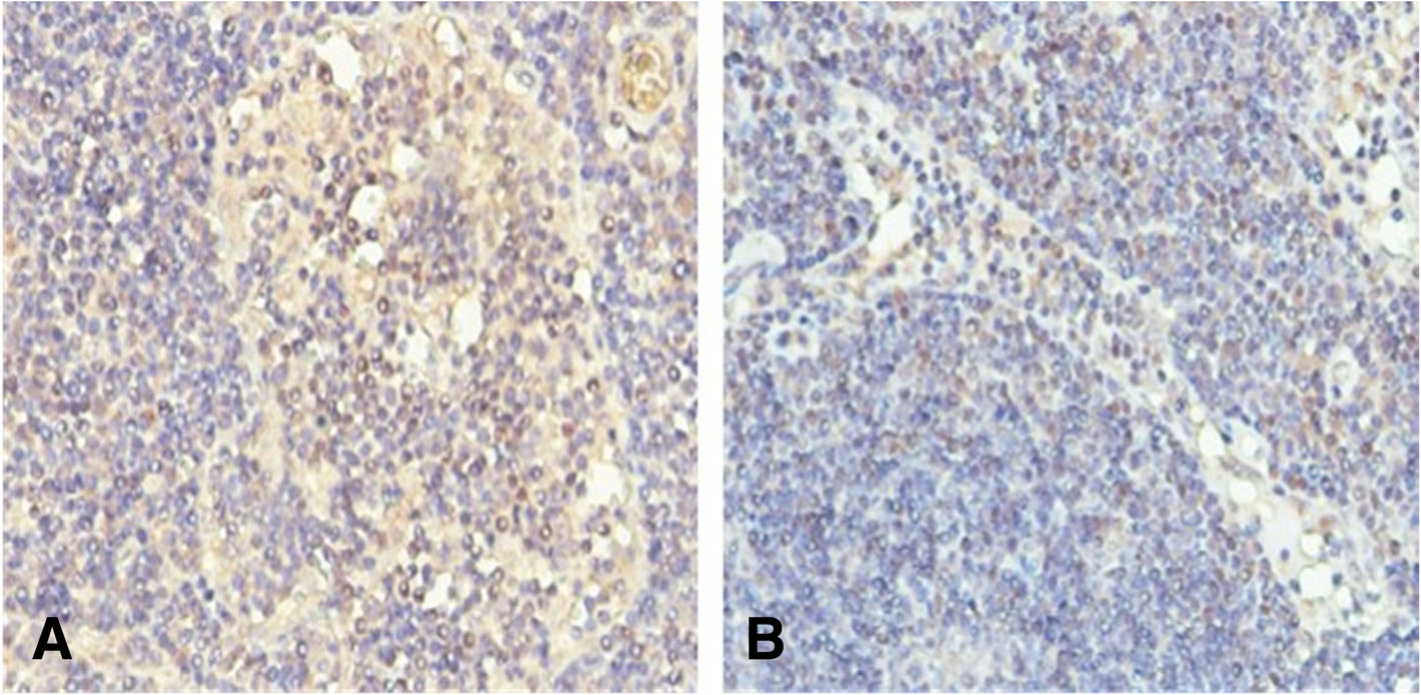
Expression of THSD7A in lymph nodes. THSD7A positive in two patients’ metastatic lymph nodes  (**a**, metastatic lymph nodes  from a patient with colorectal cancer and proteinuria; **b**, metastatic lymph nodes from a patient with colorectal cancer and without proteinuria)

Meanwhile, all lymph nodes of 2 patients with lymph node metastasis were detected THSD7A expression with immunohistochemical staining. One patient had proteinuria and the other patient didn’t. The results showed that the metastatic lymph nodes in the two patients were THSD7A positive (Fig. 2).

### Clinical follow-up of THSD7A-ab positive patients with malignant tumor

Of the 11 patients with positive proteinuria, 4 had repeated urine analysis during their follow-up period of 3 to 6 months after surgery. Three patients had complete remission of proteinuria without any specific treatment. One patient had no remission of proteinuria, but the patient was only followed up once at the third month after the surgery (Table 5).

**Table 5:** Follow-Up of THSD7A-Ab positive patients

Patients	Urinary protein	Prognosis	Follow-up time	Remission time	lymphatic metastasis
A	1+	Not remission	3 months	–	No
B	1+	Remission	6 months	3rd month	No
C	1+	Remission	3 months	2nd month	No
D	1+	Remission	6 months	4th month	Yes

*No* no lymphatic metastasis, *Yes* lymphatic metastasis

## Discussion

THSD7A was a membrane-associated N-glycoprotein with the function of promoting endothelial cell migration during angiogenesis, and it might be related to cell adhesion, growth, differentiation, proliferation and apoptosis [11, 12]. Previous studies had indicated that angiogenesis played an important role in the development of tumors [11, 13, 14]. Recently a study indicated that the expressing quantity of THSD7A was related to the clinical stages and differentiation degrees of numerous cancers [15, 16]. It suggested that THSD7A might be involved in development of various kinds of cancers.

Our experiments confirmed that THSD7A was expressed in colorectal cancer and breast cancer tissues, and the positive rates of THSD7A were 97.5% in colorectal cancer and 100% in breast cancer respectively, which were much higher than the positive rates detected in tumor tissue microarrays by Stahl and his colleagues [16]. However, while further confirmed by immunohistochemical staining in tumor tissue sections in the study, they found that the positive rate of THSD7A was 100% in high grade group and 91.7% in low grade group in colorectal cancer entities [16]. Meanwhile, the results showed that THSD7A was strongly positive in colorectal cancer and moderately positive in breast cancer, indicating that the THSD7A-positivity was expressed in different type of cancers with different patterns.

MN was the most common cause of nephrotic syndrome in adults, approximately 75% of the cases were IMN. With the discovery of autoantigen neutral endopeptidase, PLA2R, THSD7A, etc., IMN was considered as an autoimmune disease. The exposure of antigens on podocyte leads to formation of an immune complex which deposits on the basement membrane and activates the complement system, then the basement membrane and the podocyte was injured [3, 17–19]. THSD7A was the newly identified pathogenic antigen of MN, and the location in the glomerulus, structure, and pathogenicity of THSD7A was similar to PLA2R [1, 20]. But it’s difficult to explain phenomenon that there was a high prevalence of THSD7A expression in cancers, and there was a high prevalence of cancer in MN patients with THSD7A positive [20].

In order to explore the relationship among THSD7A, tumor and MN, this study firstly explored the relationship between THSD7A and proteinuria in two types of cancers. The results showed that about 10.9% of the cancer patients had proteinuria, which is higher than that was reported (9.4%) in Chinese adults from a cross-sectional survey in 2012 [21]. The prevalence of albuminuria (10.9%) was very high while considering that we had already excluded the patients with previous chronic kidney diseases, diabetes mellitus, hypertension or autoimmune diseases, infection of HBV, HCV or HIV; patients with urinary tract infection, calculi or neoplasms; as well as patients with acute kidney injury. It demonstrated that cancer patients with THSD7A positive were prone to have urinary protein, and THSD7A might be a link between cancers and proteinuria.

Four patients were followed up after surgery, 3 of them had complete remission of urinary protein without any specific intervention for proteinuria. It also supported the relationship between cancers and proteinuria, and complete removal of the cancer could induce clinical remission of proteinuria [22] We hypothesized that THSD7A might be a tumor antigen released by cancer tissues, the immune system recognized it and produced high-affinity antibodies, which bind to THSD7A on podocyte in situ and formed subepithelial immune complex, then led to proteinuria.

The significance of this study was that we demonstrated there would be high prevalence of THSD7A positive in colorectal and breast cancer. It would be related to the development of proteinuria in those patients. The results let us to think about the definition of THSD7A associated-MN. The positive rates of THSD7A were 97.5% in colorectal cancer and 100% in breast cancer respectively, so it’s hard to find a match-paired control group of neoplastic patients with negative expression of THSD7A. The study was a retrospective study, so we didn’t have detailed clinical data and more follow-up data, and renal biopsy wasn’t conducted in these patients. In further study, it would be very interested that we simultaneously detected the expression of PLA2R and THSD7A, and explored the relationship between MN and PLA2R/THSD7A in patients with malignancies.

## Conclusion

THSD7A was expressed in colorectal cancers and breast cancers with high prevalence and might play an important role in the development of proteinuria in patients with cancers. But its exact pathophysiological role in patients with MN and malignant tumors needs further studies.

## Additional files

Additional file 1: **Table S1.** Detailed information about patients with positive urinary protein M, male; F, female. **Table S2.** Correlation between urinary protein and THSD7A staining intensity of in colorectal cancer group**.** *P* value is obtained from rank sum correlation of urinary protein withTHSD7A intensity. **Table S3.** Correlation between urinary protein and staining area of THSD7A in colorectal cancer group**.** P value is obtained from rank sum correlation of urinary protein with THSD7A staining area. **Table S4.** Correlation between urinary protein and THSD7A staining intensity in breast cancer group**.** P value is obtained from rank sum correlation of urinary protein withTHSD7A intensity. **Table S5.** Correlation between urinary protein and THSD7A staining area in breast cancer group**.** P value is obtained from rank sum correlation of urinary protein with THSD7A staining area. (DOCX 17 kb)

Additional file 2: **Figure S1.** The classification criteria for histochemical staining intensity. A-C is the three categories of THSD7A staining intensity in colorectal cancer tissues: A, 3+; B, 2+; C, 1+. D-F is the three categories of THSD7A staining in breast cancer tissues: D, 3+; E, 2+; F, 1 + . (DOCX 2663 kb)

## Data Availability

The datasets used and/or analyzed during the current study are available from the corresponding author on reasonable request.
