# Supplementary material for: Expression of THSD7A in neoplasm tissues and its relationship with proteinuria
Source: BMC Nephrol. 2019 Aug 23;20:332. doi: 10.1186/s12882-019-1489-5 (PMC6708223; doi:10.1186/s12882-019-1489-5)
Supplement: Supplementary file 1 — Table S1. Detailed information about patients with positive urinary protein M, male; F, female. Table S2. Correlation between urinary protein and THSD7A staining intensity of in colorectal cancer group. P value is obtained from rank sum correlation of urinary protein withTHSD7A intensity. Table S3. Correlation between urinary protein and staining area of THSD7A in colorectal cancer group. P value is obtained from rank sum correlation of urinary protein with THSD7A staining area. Table S4. Correlation between urinary protein and THSD7A staining intensity in breast cancer group. P value is obtained from rank sum correlation of urinary protein withTHSD7A intensity. Table S5. Correlation between urinary protein and THSD7A staining area in breast cancer group. P value is obtained from rank sum correlation of urinary protein with THSD7A staining area. (DOCX 17 kb) [file 12882_2019_1489_MOESM1_ESM.docx]

**Additional files**

**Table S 1. Detailed information about patients with positive urinary protein.**

|  | Colorectal cancer | Breast cancer |
| --- | --- | --- |
| Gender (male, %) | 9/9(100%) | 0/2(0) |
| Age (years) | 58.1±10.3 | 53.0±15.6 |
| Lymph node metastasis (Cases, %) | 2/9(22%) | 1/2(50%) |

**Table S 2. Correlation between urinary protein and THSD7A staining intensity of in colorectal cancer group**

| Urinary |  | THSD7A |  |  |
| --- | --- | --- | --- | --- |
| protein | 3+ | 2+ | 1+ | 0+ |
| 3+ | 1 | 0 | 0 | 0 |
| 2+ | 3 | 1 | 0 | 0 |
| 1+ | 6 | 2 | 0 | 0 |
| 0+ | 52 | 12 | 2 | 0 |
| P |  |  |  | 0.826 |

P value is obtained from rank sum correlation of urinary protein withTHSD7A intensity.

**Table S 3. Correlation between urinary protein and staining area of THSD7A in colorectal cancer group**

| Urinary |  | THSD7A |  |  |
| --- | --- | --- | --- | --- |
| protein | 3+ | 2+ | 1+ | 0+ |
| 3+ | 0 | 1 | 0 | 0 |
| 2+ | 4 | 0 | 0 | 0 |
| 1+ | 7 | 1 | 0 | 0 |
| 0+ | 63 | 3 | 0 | 0 |
| P |  |  |  | 0.421 |

P value is obtained from rank sum correlation of urinary protein with THSD7A staining area.

**Table S 4. Correlation between urinary protein and THSD7A staining intensity in breast cancer group**

| Urinary |  | THSD7A |  |  |
| --- | --- | --- | --- | --- |
| protein | 3+ | 2+ | 1+ | 0+ |
| 3+ | 0 | 0 | 0 | 0 |
| 2+ | 0 | 1 | 0 | 0 |
| 1+ | 1 | 3 | 0 | 0 |
| 0+ | 6 | 8 | 1 | 0 |
| P |  |  |  | 0.557 |

P value is obtained from rank sum correlation of urinary protein withTHSD7A intensity.

**Table S 5. Correlation between urinary protein and THSD7A staining area in breast cancer group**

| Urinary |  | THSD7A |  |  |
| --- | --- | --- | --- | --- |
| protein | 3+ | 2+ | 1+ | 0+ |
| 3+ | 0 | 0 | 0 | 0 |
| 2+ | 1 | 0 | 0 | 0 |
| 1+ | 3 | 1 | 0 | 0 |
| 0+ | 13 | 2 | 0 | 0 |
| P |  |  |  | 0.439 |

P value is obtained from rank sum correlation of urinary protein with THSD7A staining area.
