# Supplementary material for: Expression of THSD7A in neoplasm tissues and its relationship with proteinuria
Source: BMC Nephrol. 2019 Aug 23;20:332. doi: 10.1186/s12882-019-1489-5 (PMC6708223; doi:10.1186/s12882-019-1489-5)
Supplement: Supplementary file 2 — Figure S1. The classification criteria for histochemical staining intensity. A-C is the three categories of THSD7A staining intensity in colorectal cancer tissues: A, 3+; B, 2+; C, 1+. D-F is the three categories of THSD7A staining in breast cancer tissues: D, 3+; E, 2+; F, 1 + . (DOCX 2663 kb) [file 12882_2019_1489_MOESM2_ESM.docx]

**Figure S1.** The classification criteria for histochemical staining intensity


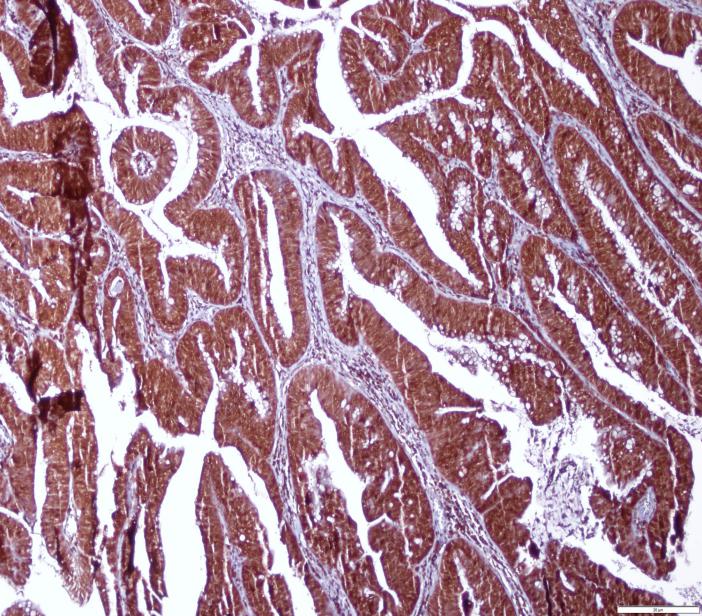

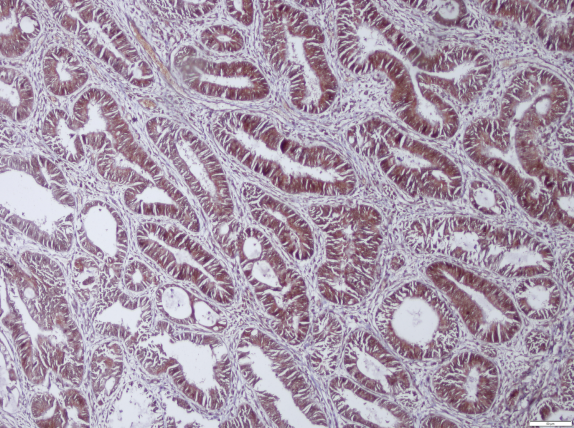

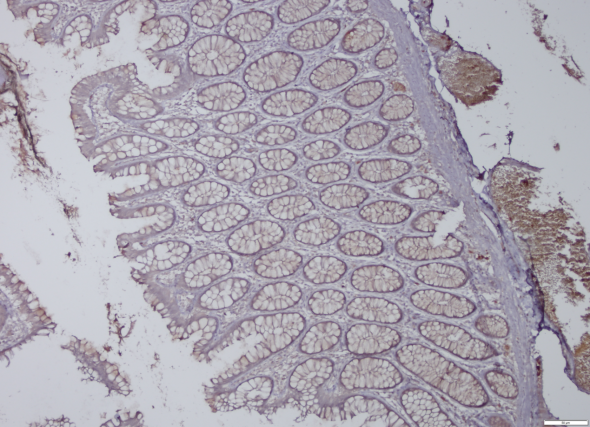


**C**

**B**

**A**


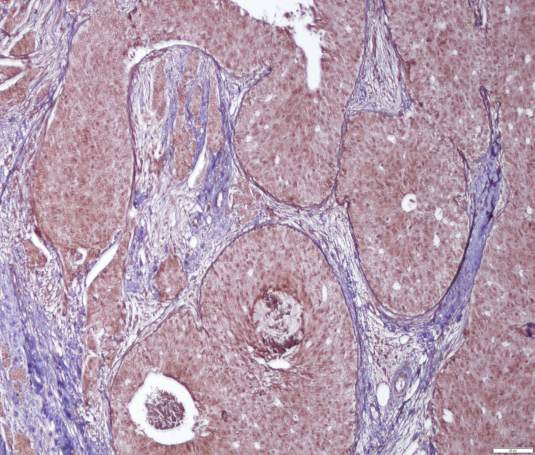

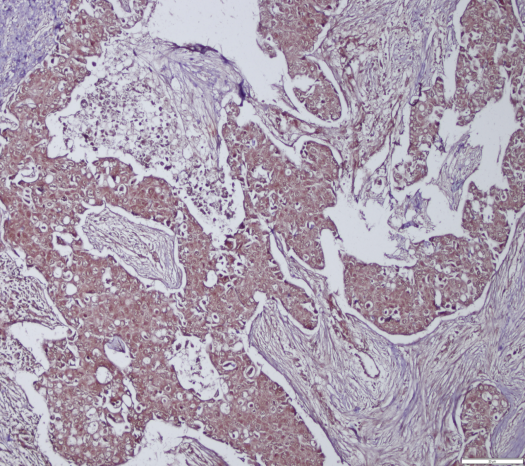

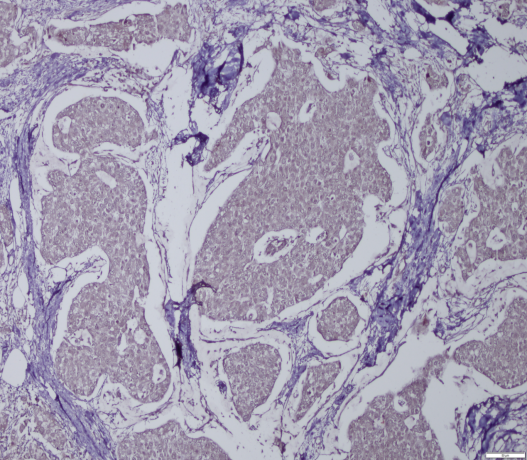


**F**

**E**

**D**

A-C is the three categories of THSD7A staining intensity in colorectal cancer tissues: A, 3+; B, 2+; C, 1+. D-F is the three categories of THSD7A staining in breast cancer tissues: D, 3+; E, 2+; F, 1+.
